# Recognizing Context-Aware Human Sociability Patterns Using Pervasive Monitoring for Supporting Mental Health Professionals

**DOI:** 10.3390/s21010086

**Published:** 2020-12-25

**Authors:** Ivan Rodrigues de Moura, Ariel Soares Teles, Markus Endler, Luciano Reis Coutinho, Francisco José da Silva e Silva

**Affiliations:** 1 Laboratory of Intelligent Distributed Systems (LSDi), Federal University of Maranhão, 65080-805 São Luís, Brazil; ariel.teles@ifma.edu.br (A.S.T.); luciano.rc@ufma.br (L.R.C.); fssilva@lsdi.ufma.br (F.J.d.S.e.S.); 2 Federal Institute of Maranhão, 65570-000 Araioses, Brazil; 3 Department of Informatics, Pontifical Catholic University of Rio de Janeiro, 22453-900 Rio de Janeiro, Brazil; endler@inf.puc-rio.br

**Keywords:** mental health, pervasive computing, context awareness, sociability, social behavior, sociability pattern

## Abstract

Traditionally, mental health specialists monitor their patients’ social behavior by applying subjective self-report questionnaires in face-to-face meetings. Usually, the application of the self-report questionnaire is limited by cognitive biases (e.g., memory bias and social desirability). As an alternative, we present a solution to detect context-aware sociability patterns and behavioral changes based on social situations inferred from ubiquitous device data. This solution does not focus on the diagnosis of mental states, but works on identifying situations of interest to specialized professionals. The proposed solution consists of an algorithm based on frequent pattern mining and complex event processing to detect periods of the day in which the individual usually socializes. Social routine recognition is performed under different context conditions to differentiate abnormal social behaviors from the variation of usual social habits. The proposed solution also can detect abnormal behavior and routine changes. This solution uses fuzzy logic to model the knowledge of the mental health specialist necessary to identify the occurrence of behavioral change. Evaluation results show that the prediction performance of the identified context-aware sociability patterns has strong positive relation (Pearson’s correlation coefficient >70%) with individuals’ social routine. Finally, the evaluation conducted recognized that the proposed solution leading to the identification of abnormal social behaviors and social routine changes consistently.

## 1. Introduction

Mental health refers to the psychological, social, and emotional well-being, so influencing our behaviors, feelings, and thoughts. Mental well-being contributes to individuals perceive their skills, work productively, contribute to their community, interact with other people, and recover from their daily routine stresses [1]. Mental disorder is a term used to describe mental health problems, such as depression, schizophrenia, and social anxiety. These disorders are responsible for affecting aspects such as mood, sleep, personality, thoughts, and social relationships [2]. Mental disorders are a health problem prevalent in a large part of the world population, affecting about 700 million people worldwide [3]. Depression is a mental disorder that affects more than 300 million people worldwide, while around 800,000 people commit suicide each year [4]. Therefore, it is possible to recognize that the prevalence of mental health problems has reached a significant part of the world population.

Mental disorders cause behavioral change that represents a relevant indicator of their onset, presence, or development. These behavioral changes are situations of interest to mental health professionals since they are used as a basis for performing assessments and interventions. In particular, social behavior changes can represent relevant indicators of mental disorders as individuals’ sociability has significant implications for their state of well-being [5]. Social relationships’ characteristics can represent aspects capable of protecting or contributing to the development of mental disorders. For example, there is evidence that social support is a relevant factor for mental health [6,7], since there is a higher likelihood of depression among people who do not have social support. There is also evidence that social isolation is associated with mental health problems, such as depression, anxiety, and suicidal ideation [8]. Moreover, social isolation imposed to reduce the rate of contagion by the COVID-19 coronavirus may further impact global mental health [9]. Therefore, symptoms of mental disorders can be externalized through changes in social behaviors, so characterizing a situation of interest for monitoring mental health.

Traditional methods of evaluating social behavior performed by mental health professionals are based on clinical evidence and information self-reported by the patient [10]. These approaches generally use retrospective reports of social experiences lived by individuals in their daily lives, in which memory time can be days, weeks, and even months. As a result, cognitive biases limit these methods, hence contributing to an incoherent exposure of the lived experience [11,12]. For example, memory bias can prevent patients from reporting their feelings and behaviors accurately [11]. Social desirability bias encourages patients to hide or modify the truth of their reports to achieve socially desirable results [12]. The clinical context in which mental health assessments occur is also a limitation since it does not represent the patients’ natural environment, implying a low ecological validity of traditional mental health assessment methods.

Currently, ubiquitous devices (e.g., smartphones, smartwatches, smart bands, and fitness bracelets) represent a promising means of mitigating those limitations [13]. The pervasive nature of these devices combined with a large amount of behavioral data from their sensors make ubiquitous computing a natural option to incorporate new system proposals for monitoring social behaviors related to mental health. Among the methodologies in this research area, the approach called Digital Phenotyping stands out. The term Digital Phenotyping refers to “moment-by-moment quantification of the individual-level human phenotype in-situ using data from smartphones and other personal digital devices” [14]. The goal of digital phenotyping is to learn and monitor patterns overtime that characterize behaviors of individuals (e.g., physical activities performed, their social interactions and mobility), based on context data derived from mobile, wearable, and Internet of Things (IoT) computing devices [13]. By using this concept, it is possible to create computational mechanisms able to perform continuous and discrete detection of individuals’ social behaviors [15]. These mechanisms can integrate computerized systems of Ecological Momentary Assessment (EMA) and Ecological Momentary Intervention (EMI), which allow mental health professionals to collect daily behavioral information from their patients and perform interventions in their natural environment. These solutions contribute to the effectiveness of the treatment and provide real-time support to the patients’ daily life.

The current literature presents solutions that use pervasive devices to recognize social behaviors related to mental health [13,16,17]. These solutions usually aim to make association, detection, classification, and prediction of mental states through features of the identified social situations [17]. However, there is still a need to develop solutions capable of recognizing sociability patterns representing the patients’ social routine, so providing a valuable tool for assessing social behavior. Consequently, it is also essential to develop solutions to monitor changes in a patient’s sociability pattern because these behavioral changes can mean the manifestation of mental disorders.

Given the need to objectively monitor social behavior, this study proposes a solution for processing social activity derived from pervasive devices to detect context-aware sociability patterns and social behavior changes. The proposed approach is able to perform incremental learning of context-aware sociability patterns through the combination of Frequent Pattern Mining (FPM) [18] and Complex Event Processing (CEP) [19]. Specifically, our proposed solution detects the time intervals in which social activities habitually occur. The recognition of sociability patterns is performed for specific contexts (e.g., weekdays, rainy days, and weekends), which enables the identification of behavior variability in different context conditions. The proposed solution is also able to identify changes in sociability patterns that reflect abnormal social behaviors and variations in social routines.

This article is an extended version of [20], where we outlined our approach to detect sociability patterns, but we did not present the solution for identifying changes in social behaviors. This paper has the following contributions: (i) we present an update of the formalization of the algorithm to detect context-aware sociability patterns; (ii) we introduce a solution for recognizing abnormal social behaviors and social routine changes; (iii) we use fuzzy logic to model knowledge of the mental health specialist needed to recognize social behavior changes; (iv) we evaluate the ability of the sociability patterns identified by the proposed solution to explain and predict users’ social behaviors; and (v) we present an extensive analysis to evaluate the social behavior change detection solution.

The remaining of the paper is organized as follows. Section 2 discuss the related works. Section 3 presents the proposed solution to detect context-aware sociability patterns and changes in social behavior. Section 4 exposes an experimental evaluation of the proposed solution using a real-world data stream. In the end, we drive our conclusions and future works in Section 5.

## 2. Related Work

Several studies have proposed solutions to identify social situations through mobile and wearable devices to support mental health professionals [17]. In particular, studies have developed solutions to transform contextual data into sociability information. We categorize these studies according to their primary objectives [17]: solutions that aim to classify, predict, or associate social features to mental state, and solutions that focus on detecting and quantifying sociability levels. Table 1 presents the related works categorized by their primary objectives.

### 2.1. Detecting and Quantifying Sociability

Some studies aim to develop solutions to identify social situations through passive detection to derive high-level information such as behavioral patterns and sociability levels. Exler et al. [21] designed a classification model capable of recognizing whether a person is alone or accompanied with an accuracy of 91.1%. This model uses location data, time of day, and activity information to perform this task. Barnett et al. [22] present a statistical approach to detect changes in sociability patterns by using phone calls and text messages, which were used to predict schizophrenic relapses. Harari et al. [23] identify patterns of stability and changes in social behavior (i.e., daily duration of conversations) of a student group over ten weeks. Bonilla et al. [24] found a set of patterns related to intensity functions of all interactions in which patients were involved by analyzing data from the use of their phone calls and social applications.

Studies also aimed to explore the passive detection of social situations to quantify sociability. Eskes et al. [25] propose the use of context data produced by smartphones (e.g., call logs, GPS locations, and Bluetooth encounters) to capture social communication and social exploration (e.g., mobility patterns and social density). These social behaviors were used to develop a statistical approach able to generate a sociability score, in which higher scores represent greater social engagement. Wahle et al. [26] monitors participants’ involvement in device-mediated communication (i.e., call logs and text messages) to quantify their sociability. Additionally, this solution recommends social exercises based on information on the intensity of social activities, time and location. Lane et al. [27] present a mobile application called *BeWell*, which has a classifier able to infer the human voice through microphone audio. This application calculates a sociability score by applying a linear regression on the total duration of conversations. In addition, *BeWell* provides feedback on the social engagement level of its users.

### 2.2. Associations between Mental State and Social Features

Researchers also work on correlating social features (e.g., duration of phone calls, frequency of using social applications) with mental states (e.g., bipolar disorder, anxiety, depression). Mental states are typically identified using clinically validated self-report questionnaires. In general, researchers use correlation coefficients, as the Pearson and Spearman correlation coefficients [41,42], to calculate the degree of association between these variables.

In a study involving university students, Wang et al. [35] recognize that social routines (i.e., conversations and Bluetooth co-locations) of students presented correlations with their depression symptoms and stress levels. Results indicate that students who had lower frequencies and shorter duration periods in their daily social interactions also had higher levels of depression and stress. Chow et al. [36] identify temporal associations between depression, affective state and social anxiety with social isolation (i.e., home stay duration measured by GPS data). Boukhechba et al. [37] demonstrate that the social roles of visited places and communication patterns (i.e., phone calls and text messages) of the students show consistent associations with depressive states and social anxiety.

Some studies focus specifically on investigating association of social features with participants’ stress levels. Wu et al. [30] found significant correlations between student stress levels with social features extracted from their social encounters measured through Bluetooth co-locations. Ono et al. [38] use a wearable device equipped with an infrared sensor to identify face-to-face interactions. This study found relationships between participants’ stress levels with frequency, duration, and number of people involved in the social interactions. To do so, evaluation and validation methods were used to measure the performance of social models.

Some studies have as the main topic of social anxiety. Gong et al. [39] performed an association between participants’ social anxiety levels and their physical behaviors, which were based on accelerometer-tracked body movement during device-mediated social interactions such as phone calls and text messages. Also, this study investigates whether the location where users performed technology-mediated communication influenced the social anxiety levels. Results indicate that people with higher levels of social anxiety exhibit more movement variations when making phone calls, especially in unfamiliar environments.

Other studies aim to correlate participants’ social activities with their mood status. Servia-Rodriguez et al. [33] found associations between sociability patterns measured by analyzing phone calls and text messages of a large number of participants with their self-reported mood assessments. Matic et al. [40] found associations between time spent on speech activities (i.e., participation in verbal social interactions) and changes in positive affects.

### 2.3. Classifying and Predicting Mental State

In this study category, researchers design solutions capable of classifying and predicting mental states. Specifically, these approaches train machine learning algorithms from social features.

Different social models have developed to classify mental states of individuals. Gu et al. [28] developed a wearable device equipped with a microphone capable of automatically identifying and analyzing paralinguistic features (e.g., Brightness_sp and MFCC5_sp) contained in the human voice during social interactions. These features were used to train the K-Means algorithm to classify the participants’ anxiety level, which obtained an accuracy of 72.73%. Chen et al. [29] use the transfer learning technique [43] to identify autism symptoms through the analysis of speech features extracted from microphone data of the wearable device.

Solutions presented by the studies also developed social models to predict mental states of individuals. For this, Wu et al. [30] use features extracted from physical social interactions identified by smartphone Bluetooth encounters to train the Random Forest algorithm, which was able to predict participants’ stress levels. Barnett et al. [22] developed and applied a statistical approach to recognize changes in patients’ communication patterns. The proposed method can predict schizophrenic relapses at two weeks in advance.

Some solutions recognize other human behaviors (e.g., sleep, mood, mobility) combined with sociability to design solutions that can identify mental states. Researchers identified several types of behaviors that have implications for mental health, allowing them to design more appropriate features to develop mental state classification models and predictions. For example, these multimodal features are used to design machine learning models to identify and predict patients with depression [26,31], bipolar disorder [32,34], and mood states [33]. Therefore, these solutions represent potential tools for supporting digital phenotyping of mental health as they recognize and utilize various patient behaviors to perform this task.

### 2.4. Discussion

Related works aim to make association, detection, classification, and prediction about mental health. From the analysis of these studies, we identified some open issues about applying passive detection of social situations to support mental health professionals. First, it is necessary to develop solutions able to identify sociability patterns that represent information about social routine of individuals. Second, analyses of such solutions should consider contextual information to identify social situations. Finally, there is a need for solutions that can detect social behavior changes to allow specialized professionals to investigate whether there is a relationship between the identified change and the patient’s mental state.

Although the related works aim to identify social behaviors to support mental health monitoring, these studies differ from our solution, so it is a challenge to use objective comparison metrics. For example, some studies develop machine learning models to classify and predict mental states, while our solution aims to extract sociability patterns and detect behavior changes. The works [22,23,24] also propose solutions capable of detecting sociability patterns, but they differ from our solution. These works design sociability patterns to quantify the duration and frequency of social interactions, while our solution recognizes periods of the day representing individuals’ social routine. Besides, these works do not recognize sociability patterns based on contextual data (e.g., weekends, holidays, and rainy days) and do not perform incremental learning, then requiring to execute batch processing.

In comparison to related works, our research has the following contributions. First, this study does not focus on the diagnosis of a specific state or mental disorder but works on identifying situations of interest (i.e., the sociability routine) for mental health professionals. Second, the proposed solution recognizes context-sensitive sociability patterns, so enabling to distinguish normal behavioral variation from behaviors that are considered anomalies. Third, besides identifying the periods of the day when the individual generally socializes, the proposed solution can recognize unusual social routines and significant changes in the patient’s social habit. Finally, the proposed solution uses a data stream mining approach to learn from social observations continuously.

## 3. Proposed Solution

In this section, we present the solution proposed to detect context-aware sociability patterns and behavioral changes. It performs incremental learning of context-sensitive sociability patterns through the combination of FPM and CEP. FPM is a computational technique that aims to discover patterns that occur with significant frequency in different data collection types, such as relational and non-relational databases, text files, and data streams [18].

The algorithm used in our solution was proposed by Lago et al. [44], which aims to learn activity patterns in smart homes (e.g., activity sequence). We applied this algorithm to digital phenotyping of mental health through the recognition of sociability patterns. First, we present a formalization update of this algorithm through unit step functions to represent the appropriate logic to identify time intervals in which social events routinely occur. We used the formalized algorithm to implement an event processing network capable of incrementally identifying context-sensitive sociability patterns. For this purpose, we used CEP concepts [19], which provide a set of tools to process data streams efficiently, so performing tasks such as data aggregation and filtering, context partitions, data window, high-level information derivation, and pattern recognition.

The proposed solution can also detect abnormal social behaviors and changes in social routines through the application of concepts of drift identification techniques. Additionally, we use fuzzy logic to model the knowledge of the mental health specialist to detect behavior change. Finally, the developed solution provides an Application Programming Interface (API) to enable the rapid implementation of strategies to identify context-aware sociability patterns and configure behavioral changes.

**Figure 1:**
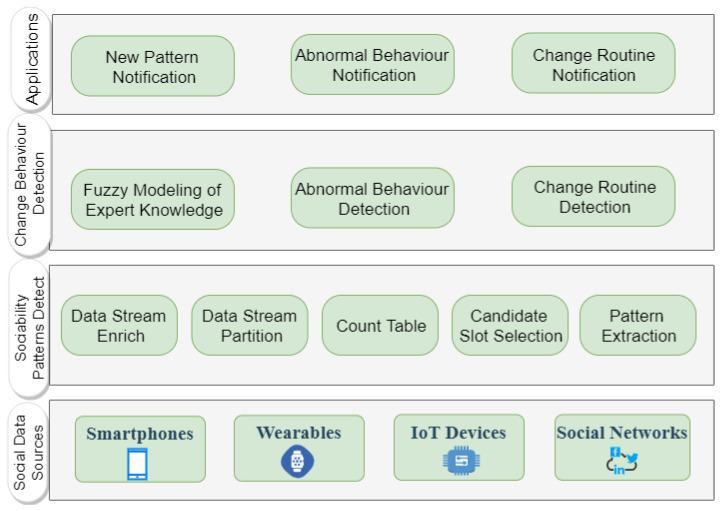
Components of the proposed solution.

Figure 1 presents an overview of the processing flow to identify context-aware sociability patterns and social behavior change. The first layer represents the generation of social events from data of online social networks and physical and virtual sensors embedded in ubiquitous devices. As examples of social events, it is possible to cite conversations identified from microphone data and interactions mediated by technology (e.g., phone calls, text messages, and social media posts). Next, the layer responsible for detecting context-aware sociability patterns supports a set of CEP rules designed to implement the algorithm to identify sociability patterns. The next layer performs tasks of detecting abnormal behaviors and routine changes. This layer also contains a Fuzzy Inference System (FIS) that models specialist knowledge needed to recognize social behavior changes. The last layer refers to client applications that receive notifications of new patterns and behavior changes emitted by the proposed solution’s components.

Ubiquitous devices (e.g., smartphones, wearable sensors, IoT devices, social networks) represent valuable social data sources. Computational methods (e.g., data mining, machine learning) can process context data from physical and virtual sensors embedded in these devices to identify social situations, such as face-to-face interactions (i.e., socialization in physical environments) and device-mediated interaction (i.e, socialization in virtual environments). For example, computational methods can process microphone and wireless communication interfaces data (e.g., WiFi, Bluetooth, NFC) to identify conversations and physical proximity [23,35]. Call logs and text messages can represent device-mediated interactions [31,33].

We emphasize that our proposed solution does not include the generation of events, but focuses on processing high-level sociability events inferred by other solutions to identify patterns and behavior changes. Next, we present in detail its components.

### 3.1. Learning Context-Aware Sociability Patterns

In this section, we present the algorithm for identifying context-sensitive sociability patterns and its implementation using CEP.

#### 3.1.1. Algorithm for Identifying Sociability Patterns

We consider that, if the social activities are detected frequently at a specific time interval, this interval composes the sociability pattern of monitored individuals. Thus, we define sociability patterns as *periods of the day in which the individual usually socializes, that is, the set of time intervals in which social activities habitually occur*. The algorithm processes the data stream to recognize time intervals [$T_{start},T_{end}$] in which the number of occurrences of social activities is higher than ϕ∗|n|. In this regard, |n| is considered as the total number of processed observations in a defined time window to model social behavior, and ϕ is a parameter to be manually set, which is responsible for indicating the sensitivity of the algorithm.

The algorithm input is a social event stream that has the start time of each social activity. The first step of the algorithm is to determine, based on the timestamp, which time frame each social event belongs. For this, the algorithm segments the time in slots with equal sizes. Each slot represents a slice of the day and has a sequential identifier. To define the size of the slot (i.e., in how many periods should divide the day), the programmer is required to specify a value for the *t* parameter, which is responsible for creating an array of counters with the total number of slots. For example, if the programmer decides to divide the day into periods of 30 min, the parameter *t* is equal to 0.5, since $\frac{24}{t}=48$ slots. This equation is responsible for creating the storage structure for counting occurrences of social activities in each slot.

After defining the size of the slots, we now describe the counting phase of the algorithm. At this stage, the algorithm uses the timestamp of each event to define its slot. By identifying the slot of social event, the algorithm increments the counter value that represents this slot in the structure responsible for storing these statistics. Therefore, when processing the flow of events, the frequency of social activities in each slot is updated. This approach of saving only the summary (i.e., the count) allows reducing the data volume since it is not necessary to memorize the full content of the events.

The next phase of the algorithm is the sociability pattern discovery, which uses the summary of the counting phase to identify frequent intervals of sociability. At this stage, it is necessary to define which slots have a sufficient number of observations, that is, a quantity that enables them to be candidate slots to form a frequent period. For this, the number counting of social observations of the analyzed slot must be greater than or equal to $S_{th}$. The algorithm uses Equation (1) to define the value of $S_{th}$. The θ parameter is entered by the programmer to set up the sensitivity of the equation.

(1) $$S_{th}=\left|n\right|*\theta *\frac{1}{\frac{24}{t}}$$

Equation (2) is responsible for iterating the slot array $C_{s}$ and verifying which slots are candidates to form a sociability pattern, so assigning zero to the count of non-candidate slots. For this, this equation defines the multiplication between the count of each slot (*slot[i]*) and the unit step function, which returns zero value in cases of negative arguments (slot[i]−slot_th<0) and one for non-negative arguments (slot[i]−slot_th>0). In the end, the slot array $C_{s}$ is sent to the process of identifying frequent sociability intervals.

(2) $$C_{s}\left[i\right]:=slot\left[i\right]*\mathrm{unit}\_\mathrm{step}\left(slot\left[i\right]-slot\_th\right)$$

Finally, after defining the requirement for a slot to be candidate, the next step is to identify which sets of slots compose an interval at which social activities are routine for the monitored individual. Equation (3) groups the adjacent non-zero candidate slots in the array $C_{s}$ into a sociability pattern. The unit step function verifies whether the sum of the event counts for the grouped slots (i.e., time intervals) subtracted from φ∗|n| results in a positive value. If this condition is satisfied, the time interval formed by these sets of adjacent slots represents a sociability pattern. In the end, the array $P_{s}$ will contain sociability patterns, that is, the time intervals in which social activities routinely occur for the monitored individual.

(3) $$P_{s}\left[i:i+n\right]:=\mathrm{unit}\_\mathrm{step}(\left(\sum_{j=i}^{i+n-1}C_{s}\left[j\right]\right)-\varphi *|n|)$$

#### 3.1.2. Context-Aware Sociability Patterns

So far, the algorithm allows identifying the individual’s sociability routine, so mapping the frequent start time of social activities. However, this context-free analysis may result in inefficiency when outlining the social habit, since the individual’s behavior may vary due to specific contexts, such as workdays, weekends, rainy days, among others. For this, we use a strategy with Context Attributes (CAs), in which several scales can be used to represent them. For example, a temporal feature may have several scales, as a broad scale, so differentiating days of week and weekends, or a more specific, so distinguishing each day of the week (e.g., Monday, Tuesday, Wednesday). We inject these CAs into the stream of social observations, which can be derived directly from event properties (e.g., timestamp) or retrieved from external sources (e.g., climate APIs). By enabling this setting, mental health professionals can define which contexts are considered more suitable for each patient and treatment.

Each CA is used as a data segmentation dimension to identify behavior change due to specific context situations. Therefore, the identification of sociability patterns is performed from a subset of data that has a particular CA. For example, all social events that occurred over the weekend (i.e., CA = Weekend) are used to identify the individual’s social routine in this context condition. The algorithm needs to create a structure (e.g., a matrix) to store slot counters for each context dimension. During the counting phase, each social event increments, in the index of its respective slot, values in the structures that store statistics for each CA of the processed social observation. In summary, we partitioned data flow based on CAs and performed incremental learning of sociability patterns for each derived data stream.

#### 3.1.3. CEP Implementation

**Figure 2:**
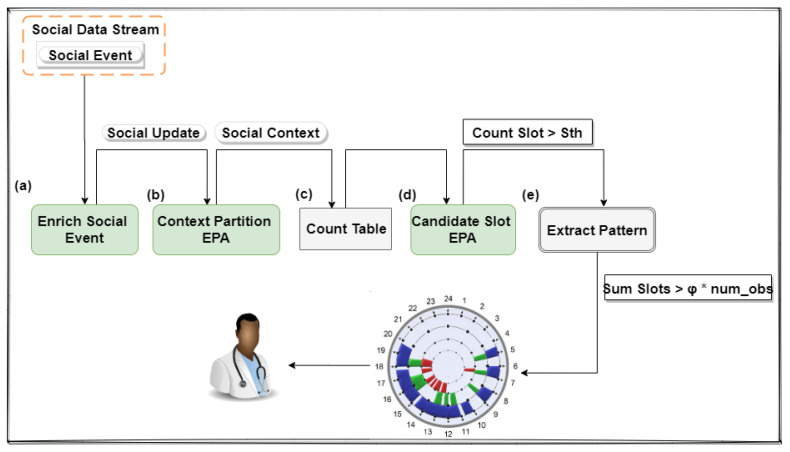
Processing workflow.

We have used CEP concepts to implement the algorithm. CEP enables to react in real time to data stream through a continuous query language [19]. This method processes data as a sequence of events, in which each event models an observation in a specific domain. Atomic events, or only events, are immutable records of something in the real world or a software system. For example, in this study, an event represents a social activity at a specific time. These social events are generated by ubiquitous devices through the processing of context data. In the implementation of the algorithm, we use *Esper* (http://www.espertech.com/esper/), which is an engine developed for CEP and continuous stream analysis. This mechanism provides an Event Processing Language (EPL) that implements and extends the Structured Query Language (SQL) standard. The processing workflow (Figure 2) consists of the following steps:

- **(a)*****Enrich Social Event*****:** it injects the slot (extracted from its timestamp) and CAs in social events. The result of this process is the emission of an enriched event named *SocialUpdate*;
- **(b)*****Context Partition EPA*****:** it segments *SocialUpdate* streams based on CAs. A derived event called *SocialContext* that has the slot and its context label is triggered for each context record of the observation;
- **(c)*****Count Table*****:** it is responsible for maintaining the count of events that occurred in specific contexts in each slot. This count is updated with each *ContextEvent* emitted;
- **(d)*****Candidate Slot EPA*****:** it is responsible for verifying which slots have reached an adequate number of observations to become candidate, so sending these to the pattern extraction phase;
- **(e)*****Extract Pattern*****:** it identifies which sets of adjacent candidate slots compose a time interval that the individual habitually socializes.

### 3.2. Social Behavior Change Detection

Another contribution of this study is an approach to recognize social routine changes and abnormal social behaviors. This approach aims to continuously detect information about changes in social habits of the monitored individual, since this information is of vital importance for treatment and monitoring, so enabling to increase the chances of effective interventions performed by mental health professionals.

#### 3.2.1. Behavioral Change Detection Strategy

We developed a strategy for detecting social behavior changes by applying the concept of drift identification techniques [45], which allows recognizing socialization flow changes of the monitored individual. Specifically, we explore unsupervised techniques to detect changes in the data stream distribution, since there is no ground truth available in social event streams to monitor performance indicators (e.g., accuracy, sensitivity, specificity, recall). Therefore, we combine the processing of data windows with a similarity metric to verify pattern change of an instant from *t1* to *t2*. It is important to note that the proposed approach detects social behavior changes for each CA, so differentiating common social routine variation from abnormal social habits.

We explored data window processing strategy provided by the Esper engine to partition data flow, so extracting sociability patterns at different time intervals. Therefore, it is possible to detect sociability patterns through current data and, subsequently, compare them with the pattern identified in a future data window to recognize occurrences of social routine changes of the monitored individual. We used the Jaccard similarity coefficient (Equation (4)) to quantify overlaps between intervals of identified sociability patterns (i.e., proportion between intersection and union of the compared patterns).

(4) $$J\left(A,B\right)=\frac{A\cap B}{A\cup B}$$

Based on the strategy described above, we developed a solution to detect two types of situations of interest for specialized mental health professionals: abnormal social behaviors and social routine changes. Abnormal social behaviors reflect a low similarity between the current sociability pattern and the individual’s social habit on a specific day. When abnormal behaviors occur with substantial frequency, it is necessary to assess whether the individual’s sociability routine has changed. Therefore, routine change is recognized when a sociability pattern extracted from a data window at time *t* has low similarity to a pattern identified from the data window *t + n*. The proposed solution maintains the first identified sociability pattern (i.e., a reference pattern) until it finds a new pattern significantly different from the reference pattern, so allowing to identify changes that happen slowly, over a long period.

**Figure 3:**
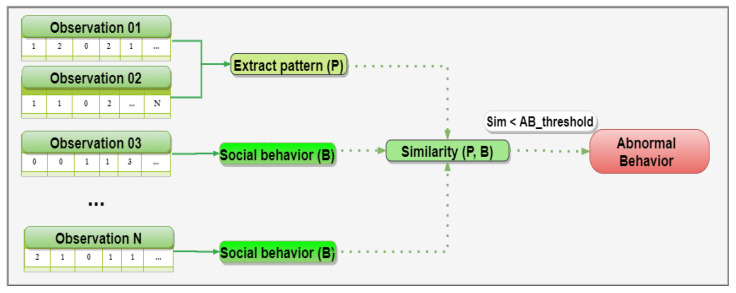
Strategy for detecting abnormal social behavior.

Figure 3 shows the strategy for detecting abnormal social behaviors. To perform this task, the specialist (e.g., a mental health professional) is required to define the time window size that models an observation of the individual’s social behavior in a given context (e.g., Mondays, rainy days, weekends). For example, in Figure 3, an observation could be composed of a one-week data window, so requiring two observations to extract a consistent sociability pattern, i.e., to conceive a predictive model of intervals of the day in which the individual habitually socializes. Therefore, to recognize abnormal behaviors, our proposed solution uses the Jaccard similarity coefficient (Equation (4)) to compare the current sociability pattern with the social behavior of the next observations. Hence, if the similarity between the current pattern and the recognized social habit (i.e., an observation) has value below a threshold defined by the specialist, the solution sends an event to notify interested parties about the identification of abnormal social behavior.

**Figure 4:**
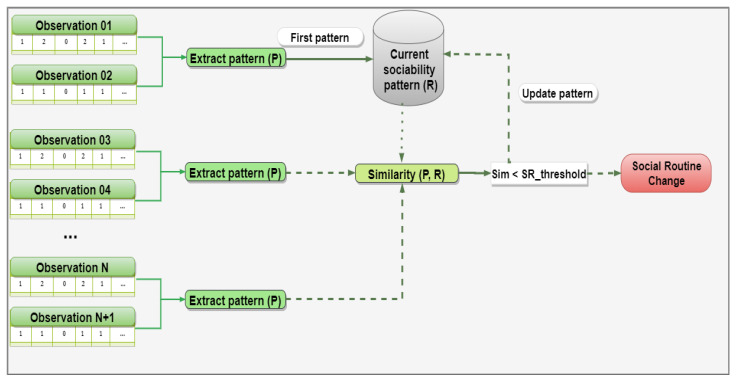
Strategy for detecting social routine changes.

Figure 4 presents the strategy for detecting social routine changes. In this scenario, sociability patterns are extracted through a determined number of observations (e.g., two observations), which represent the current social routine of the monitored individual. In principle, the solution persists in memory the first sociability pattern identified, which is used as a comparison model with the next patterns to recognize significant social routine changes. Specifically, when the similarity is below the defined threshold, two tasks are triggered to: (i) update the current sociability pattern with the most recent routine and; (ii) notify interested parties by sending a social routine change event.

#### 3.2.2. Expert Knowledge Modeling

As we could see, it is possible to identify the specialist’s need to define a similarity threshold between patterns. The specification of the change threshold is subjective, as it depends on the specialist’s knowledge, and has an imprecise essence, since a rigid limit may not adequately model the change that has occurred. For example, consider that an specialist has configured the behavior change detection solution with a similarity threshold of 60%. In this scenario, if the similarity between sociability patterns is 59.9%, abnormal social behavior is detected even though it is very close to the specified threshold. Therefore, it is necessary to define strategies that allow modeling the specialist’s knowledge automatically, so enabling to send behavior change alerts with a judgment of the belief degrees.

We implemented expert knowledge modeling through fuzzy logic concepts to consider the imprecise nature of this task. Specifically, we used the open-source library *jFuzzyLogic* (http://jfuzzylogic.sourceforge.net/) [46,47] to develop fuzzy controllers. This library uses Fuzzy Control Language (FCL), which is a domain-specific language to facilitate the development of fuzzy systems. *jFuzzyLogic* enabled the integration of FIS into the proposed solution to allow the specialist to specify linguistic variables, fuzzy sets, and rules.

The motivation for using fuzzy logic in this task is the possibility of representing imprecise and qualitative human knowledge through fuzzy sets instead of using crisp sets [48]. Fuzzy logic allows computational systems to model human reasoning so that output variables can vary from false to true gradually, then making it possible to express partially true or partially false conclusions. Another reason is the notation of defining fuzzy rules through linguistic variables (i.e., natural language) and logical connectors [49], which presents semantics that is easy to understand for users. Therefore, we used fuzzy logic due to its ability to model expert knowledge through fuzzy sets and fuzzy rules and its notation that is easy to understand. Moreover, fuzzy logic has already been used to model situations of interest to mental health professionals [50].

First, the specialist is required to determine three fuzzy sets: sensitivity, similarity, and drift. Sensitivity sets define discrepancy levels between patterns that represent a change, i.e., it models the sensitivity knowledge to detect changes. Similarity sets define correspondence levels between evaluated patterns. Finally, drift sets represent the FIS output (i.e., defuzzifier) responsible for modeling change levels in social behaviors.

**Figure 5:**
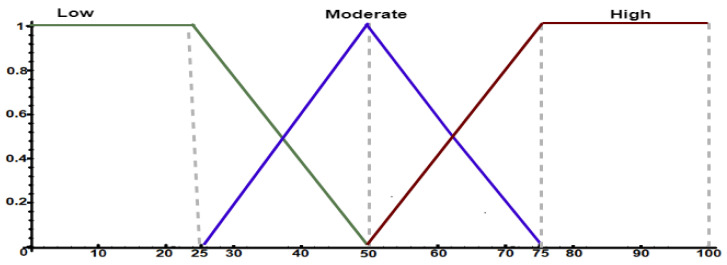
Fuzzy sets to model behavior change detection sensitivity.

After defining fuzzy sets, the next step is to subdivide them to represent specialist’s knowledge to assign linguistic terms to the intervals of each set. Therefore, the specialist should use FCL to perform this task. Code 1 shows a configuration example of the sensitivity sets using FCL, while Figure 5 shows their visual representation. The specialist divided the sensitivity input variable into three levels: low, moderate, and high. In this scenario, a certain level of intersection between intervals is observed, so representing a gradual pertinence transition.

**Figure 6:**
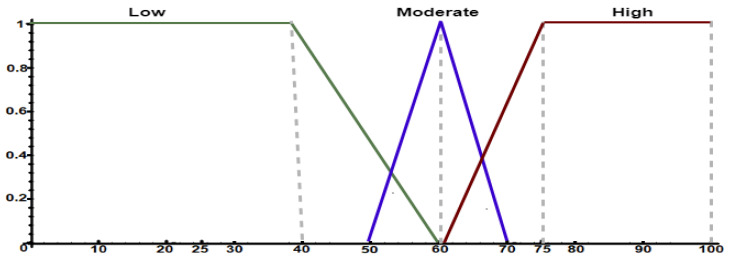
Fuzzy sets to model the similarity levels between patterns and behaviors.

The specialist is also required to represent his/her knowledge through FCL to define similarity set partitions and their respective linguistic terms. Code 2 shows a configuration example of the similarity sets using FCL, while Figure 6 shows their visual representation. The specialist subdivided the similarity input variable into three levels: low, moderate, and high. In this scenario, intervals have an intersection level to model uncertainty.

**Figure 7:**
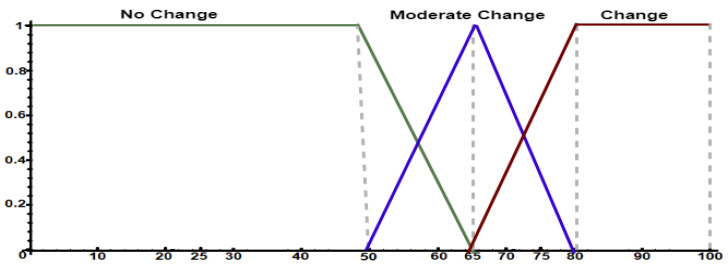
Output fuzzy sets defined to assess behavior change.

The third set to be specified is the drift, which is representing the FIS output, i.e., it models the occurrence of social behavior changes of the monitored individual. Code 3 presents a configuration example of the drift sets, while Figure 7 shows their visual representation. The specialist described three linguistic terms: no_change, moderate_change, and change. In this example, we used the Center of Gravity (COG) defuzzification method to determine the inference’s final value from activated rules. *jFuzzyLogic* supports several fuzzification and defuzzification methods.

Next, the specialist is required to specify fuzzy rules that guide the solution’s decision. These fuzzy rules have the primary structure <condition> *AND* <condition> *THEN* <consequence>. The specified fuzzy rules form the knowledge used by the FIS to determine the final value of the behavior change inference. Therefore, based on fuzzy sets presented above, the fuzzy rules contained in Code 4 can be specified using FCL.

At the end of the expert knowledge modeling process, the solution can detect social behavior changes considering uncertainty in this task. Specifically, the solution sends a JSON object that contains information such as date, context, similarity index, and the pertinence degree to each interval of the fuzzy output set. Code 5 presents an example of social behavior change notification considering the expert knowledge modeled in the previous cases. In this example, the output variable (i.e., defuzzification value) is ≈74.86, which is ≈65% contained in the interval that represents change and ≈34% in the partition that reflects moderate change.

### 3.3. Application Programming Interface

The solution designed by this study provides an API to its users, which allows them to quickly implement strategies for detecting sociability patterns and configuring the recognition of behavioral changes. Development of this API used the Builder design pattern (Constructor) [51], which enables separation of the construction of a complex object from its representation, so dividing this process into parts (i.e., steps). We mitigate the complexity inherent in the creation and instantiation of the EPLs and solution configuration using this design pattern.

Next, the user specifies the RootTopic attribute (setRootTopic) with the value ”com/lsdi/sociability”. The solution uses this attribute to publish new sociability patterns and behavior change notifications in MQTT broker, allowing interested client applications to subscribe to this topic to receive updates. Finally, the user set true value to the parameters AbnormalBehavior and ChangeBehavior (setAbnormalBehavior and setChangeBehavior) for the solution to activate the detection of abnormal behaviors and social routine changes.

Code 6 presents an example of use of the designed API. Specifically, the developer create an object called *SociabilityPattern*, which configures and enables the entire solution operation. In this example, the developer initializes the constructor with two parameters: “MONDAY_” and 50.0, so representing the CA considered and the sensitivity level to detect behavior changes, respectively. Next, the developer specifies the *RootTopic* attribute (setRootTopic) with the value “com/lsdi/sociability”. The solution uses this attribute to publish new sociability patterns and behavior change notifications in an MQTT broker [52], so allowing interested client applications to subscribe to this topic to receive updates. Finally, the developer set true value to the parameters *AbnormalBehavior* and *ChangeBehavior* (setAbnormalBehavior and setChangeBehavior) for the solution to activate detection of abnormal behaviors and social routine changes.

## 4. Experimental Evaluation

The proposed solution identifies context-aware sociability patterns using incremental unsupervised learning. Consequently, applying metrics commonly used to evaluate learning models is challenging because there is no ground truth available to compare results. By considering this, we evaluate the proposed solution’s feature to consistently recognize sociability patterns for modeling social routine and behavior changes.

In [20], we performed the following: (i) a comparison of the similarity between the sociability patterns identified with the intervals recognized by Gaussian Mixture Models (GMMs); (ii) an analysis of the contribution of context-aware sociability patterns to understand the monitored individual’s social routine. Here, we performed a more in-depth evaluation to recognize the proposed solution ability to identify context-sensitive sociability patterns that model social behavior and detect behavioral changes.

### 4.1. Data Description

We evaluated the solution using a public dataset [35], which is referred to as *StudentLife*. Wang et al. [35] performed during 66 days a passive sensing of social activities (i.e., conversations) derived from microphone data gathered from smartphones of 48 Dartmouth College undergraduate and graduate students. Conversation samples are composed of two fields: start and end timestamps of conversations experienced by participants. All collected data was anonymized to preserve privacy of the monitored individuals.

**Figure 8:**
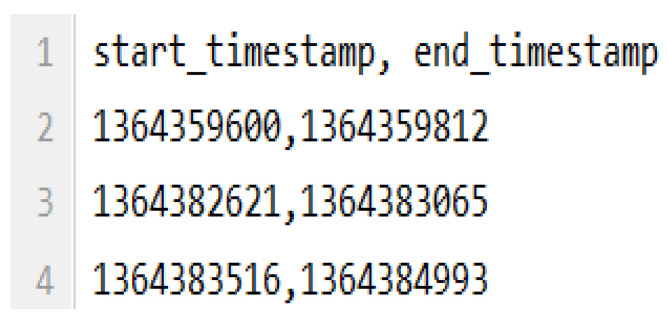
Dataset features used in the solution.

The used dataset contains conversation samples composed of two features: start and end timestamps of social interactions. Figure 8 shows the first lines of the dataset of an individual. For example, the second line in this file records that he/she experienced a conversation that started at Unix timestamp 1364359600 and ended at Unix timestamp 1364359812.

Firstly, we performed a data cleaning process to remove users who had insufficient data to conduct experiments. Only users who contained at least 52 days of collected data (≈80% of the study days) are in this experiment. We used data from 24 individuals who had sufficient data.

We represent each record as a social event to design a proper data flow for the proposed processing network. For this, we derived the following information from social activity records: social activity type (i.e., conversation), start time, and a set of CAs. The first step was to convert the Unix timestamp to a Date Java object to represent the event start time attribute and extract CAs. We identified the weekday when events occurred using their timestamps, so enabling to specify temporal context scales. We defined two context scales: a fine-grained scale composed of weekdays (e.g., Monday, Tuesday, and Wednesday) and a broad one to distinguish weekends (i.e., Saturdays and Sundays) from midweeks (i.e., Monday to Friday). In the end, the structure of the generated social event flows was as follows:


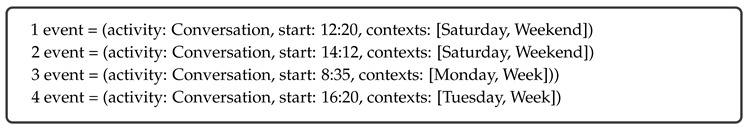


### 4.2. Experimental Design

The first evaluation consisted of verifying the ability of the context-aware sociability patterns to model and explain social behaviors, i.e., we verified whether sociability patterns could explain and predict stable social routine (i.e., individuals repeating their social behaviors over the days) and less able to explain unstable social routine. For this, we used Pearson correlation coefficient [41] to assess the association between the ability of sociability patterns to explain social routine and stability of the individual’s social routine. This coefficient measures linear correlation between two variables, which assumes values between −1 (perfect negative correlation) and 1 (perfect positive correlation). Therefore, higher levels of positive associations indicate that the proposed solution recognizes consistent sociability patterns and capable of modeling monitored people’s social behaviors.

In the end, we evaluated the proposed solution to detect social behavior changes. For this, we joined data of two users who had different social routine, hence enabling to identify the moment when the change occurs. We verified whether the proposed solution can accurately detect this change and its ability to adapt to the new sociability pattern.

### 4.3. Ability to Model Social Routines

This experiment aimed to assess the ability of the sociability patterns to model social routine. Specifically, a sociability pattern should explain the social behavior of the monitored individual, which should be correlated with his/her social habit, because the more stable the individual’s social behavior, the greater the pattern’s ability to predict it. We analyzed association between the identified sociability patterns’ prediction level and the social routine’s stability. For this reason, we used Pearson correlation coefficient for quantifying this association. By using this correlation coefficient, patterns’ ability to explain and predict social routine of the monitored individuals were identified.

We defined that a social observation consisted of a data window of one week (i.e., seven days). For example, for the *MONDAY* context, an observation consisted of data from 1 day, since a week has only 1 day with that context. Therefore, the first step of this experiment was to define the number of observations necessary to extract patterns consistent with the monitored individual’s social behavior. For this, we identified sociability patterns considering each day of the week as a CA (i.e., *MONDAY*, *TUESDAY*, *WEDNESDAY*, *THURSDAY*, *FRIDAY*, *SATURDAY*, and *SUNDAY*), and we checked the predictive performance of sociability patterns when using different numbers of social observations to design them. We defined prediction performance as the ability of sociability patterns to explain and predict individuals’ social routines.

**Figure 9:**
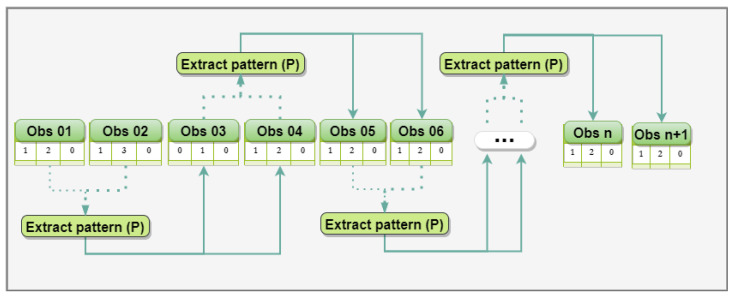
Evaluation of the prediction performance of sociability patterns using two observations.

Figure 9 presents an example of scenario for assessing the prediction performance of sociability patterns using two observations. We performed this evaluation using one, two, three, and four observations to project sociability patterns, so allowing us to compare the predictive performance of each configuration. Each execution consisted of the following steps: (i) recognizing the sociability pattern with the number of specified observations (i.e., one, two, three, or four); (ii) measuring the Jaccard similarity index between the pattern extracted with the next social observations; and (iii) identifying a new social pattern from the evaluated observations to represent a new reference pattern.

We performed the experiment for all users considering CAs specified previously (e.g., *MONDAY*, *TUESDAY*, and *SUNDAY*), so making it possible to recognize the predictive performance (i.e., the similarity between sociability patterns and observations) of sociability patterns for these CAs. We calculated the average prediction performance to identify the number of observations required to extract sociability patterns to explain social routine.

**Figure 10:**
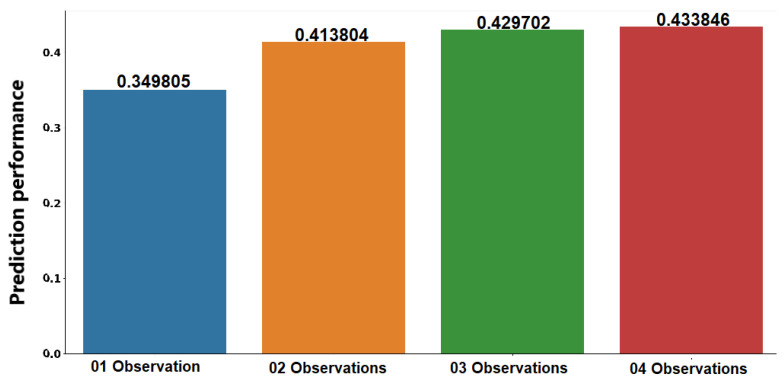
Prediction performance of sociability patterns.

Figure 10 shows the average predictive performance of the sociability patterns identified using one, two, three, and four observations. From results, we identified that extracting the sociability pattern using only one observation resulted in poor predictive performance compared to other configurations. Extraction of social patterns with two, three, or four observations showed similar predictions. Therefore, we concluded that the most appropriate approach was to use two observations to extract sociability patterns since we could identify them in less time with predictive performance similar to other configurations.

**Figure 11:**
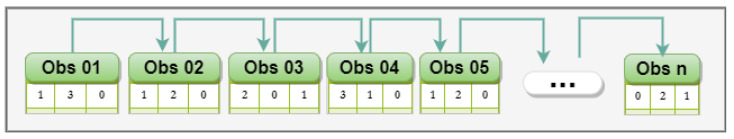
Configuration used to evaluate stability of individuals’ social routines.

After quantifying prediction levels of the extracted sociability patterns, we measured stability of the social routine of the individuals who participated in this study. We calculated average similarity between one day and the subsequent day (Figure 11), i.e., similarity of the individual’s social behavior between consecutive days. CAs considered each day of the week, similar to the previous experiment. In the end, we calculated average stability of the individuals’ social routine, so allowing us to correlate this variable with prediction levels of sociability patterns.

**Figure 12:**
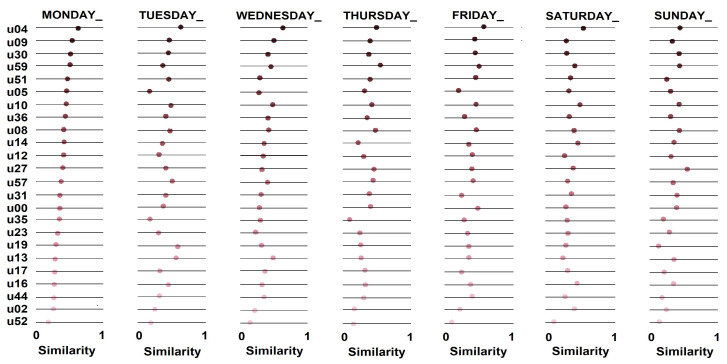
Stability of the individuals’ social routines.

We performed a stability analysis of the individuals’ social routines to recognize essential information to understand their social behaviors. Figure 12 shows the stability of the individuals’ social routines (i.e., the similarity of social behaviors between days), so making it possible to identify that most users had social habits with stability below 40%. However, some users had more stable routines, such as *u04* and *u27*. From this analysis, we expect that sociability patterns could explain and predict more consistently social behaviors of the more stable users and that present lower levels of predictions when applied to individuals with more unstable social routines.

**Figure 13:**
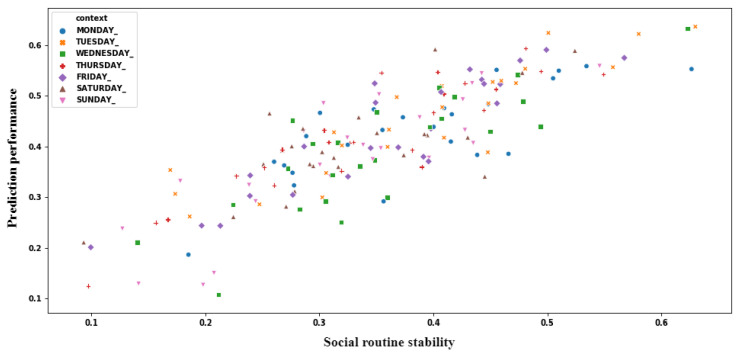
Correlation between prediction performance of sociability pattern and social routine stability.

So far, we quantified the prediction performance of the extracted sociability patterns and the stability of the individuals’ social routine. Therefore, we can perform association analysis between these two variables using Pearson correlation coefficient. Figure 13 shows this association, in which y-axis represents the average of prediction performance of the social patterns, and x-axis represents the average of the individuals’ social routine stability. When analyzing Figure 13, we can identify a clear correlation between these two variables, so representing a linear relationship. Pearson correlation coefficient resulted in +0.86, which represents a strong positive association between these variables.

**Figure 14:**
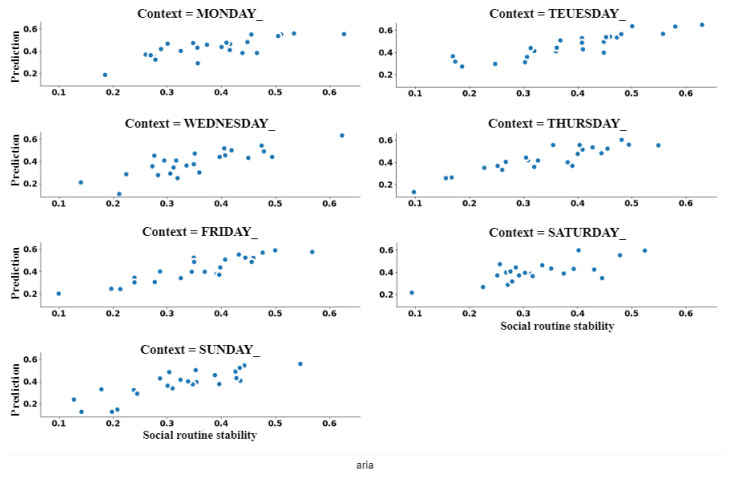
Correlation between prediction performance of sociability pattern and social routine for each Context Attribute (CA).

**Figure 15:**
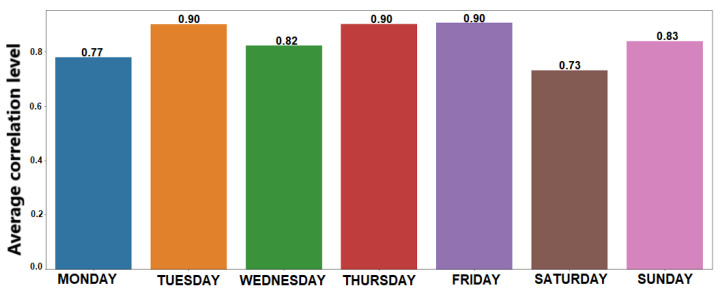
Average correlation level for each CA.

Figure 14 shows the relationship between prediction performance of the sociability patterns and stability of the individuals’ social routines for each CA, so making possible to identify a linear relationship between these variables. Figure 15 shows the result of applying the Pearson correlation coefficient between these variables for each specified CA, so indicating that association levels were higher than 0.7, which represents strong positive correlations. When analyzing these results, we can recognize that prediction performance of the extracted sociability patterns remains related to stability of the social routine in all evaluated CAs.

From this experiment, we can recognize that sociability patterns of the proposed solution satisfactorily model social routines of individuals. Therefore, sociability patterns can be used to reliably understand and predict social behavior since they have strong correlations with users’ social habits.

### 4.4. Evaluation of Social Behavior Change Detection

This experiment aimed to verify the performance of the social behavior change detection solution. In particular, we evaluate its ability to detect social behavior changes and adaption to the new social behavior of the monitored individual. Therefore, we expect that the solution to accurately identify observations that represent abnormal social behaviors and social routine changes.

Firstly, we defined a similarity threshold that represents a change in social behavior. For this reason, we calculated mean and standard deviation of the stability of social routines for all users, so defining this threshold as [μ+σ]. Similar to the configuration of the previous experiment (Figure 11), we calculated mean and standard deviation of the similarity of social behaviors of individuals between consecutive days considering each day of the week as CAs. In the end, we identified that users had an average of 35.4% of stability in their social routines and standard deviation of 10.7%. Therefore, we specified the threshold for social behavior changes at 46.1%.

We combined data from two users with significantly different social routines to simulate a change in social behavior. For this, we selected users who had more stable social habits. We identified that users u27 and u04 presented social routines with satisfactory stability for this experiment by considering results from the previous experiment.

**Figure 16:**
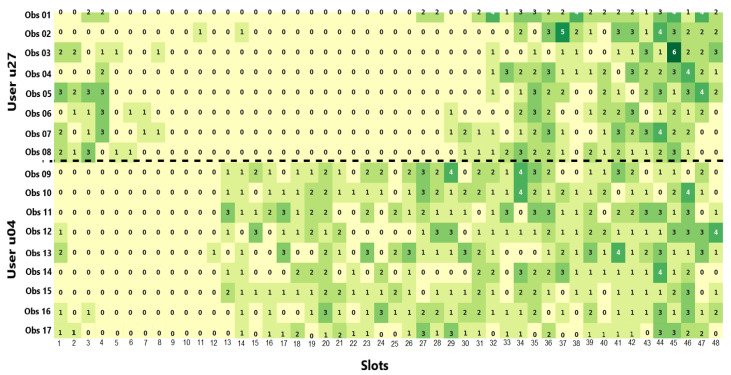
Merge of the social routines of the users u27 and u04.

Figure 16 shows social routines of the individuals u27 and u04, in which each cell contains the number of social events in a given time slot of 30 min (t = $\frac{24}{0.5}$). From this visualization, we can recognize an evident change in social behavior at the limit that separates the two users’ data. In this scenario, we expect that the proposed solution would detect abnormal behaviors when starting to process data of the user u04 and recognize the social routine change. Additionally, the solution adapt to the new pattern, then providing a new sociability pattern capable of explaining and predicting the new social routine.

Social event streams for the proposed solution were created from data of the selected users. After processing data stream, our solution detected social behavior changes presented in Table 2. These results demonstrate that it identified abnormal behaviors and social routine changes precisely. Specifically, our solution identified a high similarity between social patterns and observations (i.e., similarity > 46.1%) while processing user data u27, so not representing behavioral changes. From the first observation of the user u04, our solution started to detect abnormal social behaviors, so recognizing the social routine change by extracting the first pattern using data from this user (i.e., 5th pattern). This new reference pattern remained consistent with the next processed social observations, i.e., the solution can efficiently adapt to data stream changes.

### 4.5. Discussion and Limitations

This section presented an experimental evaluation of the proposed solution that significantly extended experimental evaluations reported in [20]. We performed an in-depth evaluation of the components designed to detect context-aware sociability patterns and behavior changes. From these experiments, we found that patterns recognized by the solution can model and predict social routines of individuals considering context information. Moreover, it can detect significant changes in social habits consistently.

In [20], we compared the similarity between social intervals detected by the proposed solution with those recognized by a batch processing algorithm. From the evaluation in [20], we recognized that the compared solutions identified sociability patterns with 86.33% similarity. We also analyzed sociability patterns based on CAs to investigate their contribution to understand social habits. We identified that context-based recognition provides insights into sociability patterns hidden in the context-free analysis. Therefore, the detection of sociability patterns based on CAs improves the understanding of social habits because it enables to distinguish abnormal behaviors from expected changes due to the context.

The aims of our experiments differ from the evaluations performed by the related works, hence it is a challenge to compare their results using specific metrics. For example, studies that design machine learning models to classify and predict mental states use metrics such as accuracy, precision, recall, whereas in our unsupervised sociability pattern learning approach, we use methods to assess the ability to model social routine and detect behavior changes. The works [22,23,24] also assess sociability patterns, but they differ from our experiments. Harari et al. [23] computed test-retest correlations between the observed behavior durations for adjacent weeks. Barnett et al. [22] analyzed the rate of anomalies in behavioral patterns based on a statistical test inspired by Filzmoser’s approach to predict schizophrenic relapse. Bonilla et al. [24] analyzed the applying of the Poisson mixture model to obtain the intensity functions of all calls in which patients were involved.

Our experiments evaluated the ability of the proposed solution to identify context-aware sociability patterns capable of explaining social routine and detecting social behavior changes. From this analysis, we identified that the prediction performance of social patterns has a strong positive correlation with the stability of the social routine (i.e., Pearson correlation coefficient greater than +0.7) in all CAs considered, so enabling to recognize that the proposed solution detects patterns consistent with the social behaviors of the monitored individuals. The evaluation of the social behavior change detection solution analyzed results of the detection processes performed by the solution when processing data containing changes in social routines. This experiment recognizes that our proposed solution can accurately detect and report abnormal social behaviors and social routine changes. Therefore, this is a promising tool for monitoring mental health, since reports of behavioral changes may indicate the onset, presence, or development of mental disorders.

This study has some limitations. First, the algorithm requires to manually enter two parameters: φ and θ. Therefore, the recognized sociability pattern depends on the predefined values chosen empirically rather than automatically setting the best values based on processed data. Second, the experimental evaluation was based on only one type of social activity (i.e., conversations). Other sources of social interactions should be considered, such as interactions on mobile social networks and telephone call communications. Third, social routine change does not necessarily imply change in sociability, as individuals can change their routine and maintain the same sociability level. However, the solution can identify social routine changes (i.e., whether there is a change in sociability), so allowing mental health professionals to interpret and investigate changes in the user’s social aspect. Finally, another aspect is the homogeneous essence of the study participants (i.e., university students). Our solution should be validated with a more heterogeneous population, especially mental health professionals and their patients.

## 5. Conclusions and Future Work

This work presented an approach for monitoring mental health through awareness of the social situation. Specifically, we introduced a solution based on FPM and CEP concepts to recognize intervals of the day when a monitored individual habitually socializes for each contextual condition. We also presented the solution developed to recognize abnormal behaviors and routine changes. Additionally, we introduced specialized knowledge modeling through fuzzy logic to allow the solution to send notifications of behavioral changes considering the imprecision of this task. From the evaluation conducted, we demonstrate that the predictive performance of context-aware sociability patterns has a strong correlation with social routine stability and that the solution can detect social behavior changes. We conclude that our proposed solution can be integrated into mental health monitoring tools to objectively collect patterns and changes in social behaviors, then providing support to mental health professionals and contributing to the effectiveness of the treatment proposed for patients.

As future work, we plan to address some open issues. The first one is to detect sociability patterns previously specified by mental health professionals, so enabling to identify situations of interest. We would also like to create dashboards for sociability patterns in an appropriate way for professionals, so facilitating analysis of social behavior. Another task is to update the solution to add information about the sociability level (e.g., social interaction intensity) to extracted patterns and identified behavioral changes. We also intend to develop an approach capable of automatically defining the best values for the parameters of the algorithm. Moreover, we plan to extend our solution to detect patterns related to other behaviors, such as physical activity and mobility. Plans also include validating our solution with professionals and their patients.

## Figures and Tables

**Figure:**
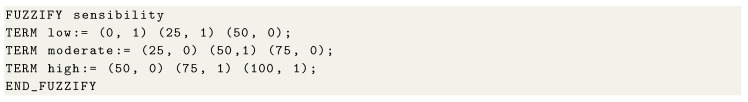
Code 1: Configuration example of the sensitivity sets using FCL.

**Figure:**
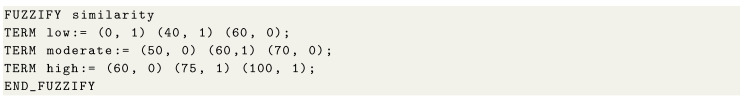
Code 2: Configuration example of the similarity sets using FCL.

**Figure:**
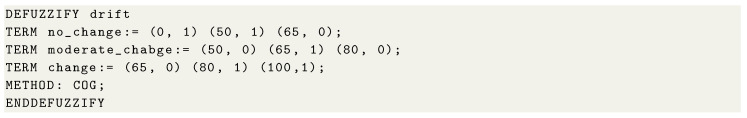
Code 3: Configuration example of the drift sets using FCL.

**Figure:**
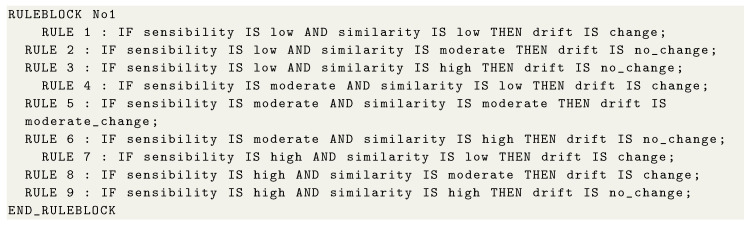
Code 4: Fuzzy rules for behavior change.

**Figure:**
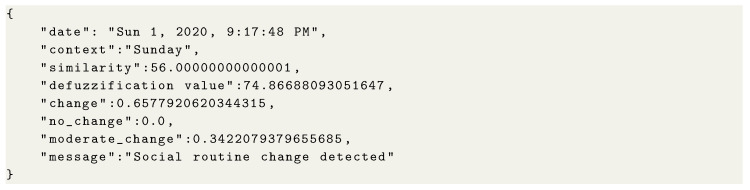
Code 5: Notification of social behavior change.

**Figure:**
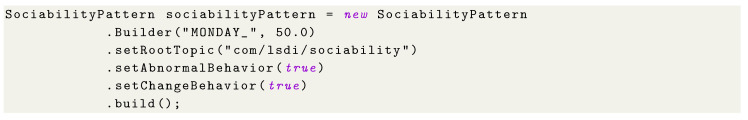
Code 6: Application Programming Interface (API) usage example.

**Table 1:** Categorization of related works.

Type of Study	Goal	References
Detection	Detecting and quantifying sociability.	[21,22,23,24,25,26,27]
Classification	Classifying a mental state through social features.	[26,28,29,30,31,32,33]
Prediction	Predicting a mental state through social features.	[22,30,32,34]
Association	Associating sociability with a mental state.	[30,33,35,36,37,38,39,40]

**Table 2:** The flow of social behavior change detection. Red lines represent the identification of abnormal behaviors and social routine change.

Observation	Uid	Similarity	Change
03	*u27*	0.520000	**Normal social behavior**
04	*u27*	0.727273	**Normal social behavior**
2nd Pattern	*u27*	0.680000	**Maintained the reference pattern**
05	*u27*	0.652174	**Normal social behavior**
06	*u27*	0.520000	**Normal social behavior**
3rd Pattern	*u27*	0.640000	**Maintained the reference pattern**
07	*u27*	0.576923	**Normal social behavior**
08	*u27*	0.555556	**Normal social behavior**
4th Pattern	u27	0.666667	**Maintained the reference pattern**
09	*u04*	0.411765	**Abnormal social behavior**
10	*u04*	0.444444	**Abnormal social behavior**
5th Pattern	*u04*	0.428571	**Social routine change detected**
11	*u04*	0.657143	**Normal social behavior**
12	*u04*	0.647143	**Normal social behavior**
6th Pattern	*u04*	0.647059	**Maintained the reference pattern**
13	*u04*	0.540541	**Normal social behavior**
14	*u04*	0.575758	**Normal social behavior**
7th Pattern	*u04*	0.600000	**Maintained the reference pattern**
15	*u04*	0.666667	**Normal social behavior**
16	*u04*	0.685714	**Normal social behavior**
8th Pattern	*u04*	0.666667	**Maintained the reference pattern**
17	*u04*	0.647059	**Normal social behavior**

## Abbreviations

The following abbreviations are used in this manuscript:

IoT	Internet of Things
EMA	Ecological Momentary Assessment
EMI	Ecological Momentary Intervention
FPM	Frequent Pattern Mining
CEP	Complex Event Processing
API	Application Programming Interface
FIS	Fuzzy Inference System
CA	Context Attribute
EPL	Event Processing Language
SQL	Structured Query Language
FCL	Fuzzy Control Language
COG	Center of Gravity
GMM	Gaussian Mixture Model
